# Negative Linear or Unimodal: Why Forest Soil Fungal Latitudinal Diversity Differs across China

**DOI:** 10.1128/spectrum.02515-22

**Published:** 2023-02-22

**Authors:** Wenchen Song

**Affiliations:** a Key Laboratory of Ecology and Environment in Minority Areas (Minzu University of China), National Ethnic Affairs Commission, Beijing, China; b College of Life and Environmental Sciences, Minzu University of China, Beijing, China; University of Mississippi

**Keywords:** forest, soil fungal diversity, fungal latitudinal diversity, fungi in China

## Abstract

To identify the reasons for the inconsistency in patterns of latitudinal gradients of forest soil fungal biodiversity in China, a reanalysis of data was performed. Causes are linked to the different environments of continents and islands and the inconsistency between different classification standards. The following three suggestions are made for future studies: sites on the mainland and islands should be distinguished in these types of studies, the Shannon index should be used to represent fungal diversity instead of operational taxonomic unit (OTU) richness, and using the diversity of higher taxa (such as family level) instead of OTU level represents a potential proxy for species-level diversity.

**IMPORTANCE** Latitudinal gradients of forest soil fungal biodiversity in China have been previously investigated; however, the results of these studies were inconsistent. In the present study, I reanalyzed the data from these studies on all forest types in China and showed that the differences in forest soil fungal latitudinal diversity were caused by the different environments of continents and islands, as well as by the inconsistency between different classification standards. Accordingly, three suggestions were outlined for future studies on this and similar topics. This study makes a significant contribution to the literature because these findings can be used to improve our understanding of the forest soil fungal latitudinal diversity and as a basis for future studies.

## OBSERVATION

Latitudinal gradients of forest soil fungal biodiversity have attracted considerable interest from ecologists (1). China covers a vast territory with a large latitudinal gradient from tropical to boreal forests. It is a key area in fungal ecogeography. Until recently, there have been six studies on the variation in fungal biodiversity of forest soils across latitudinal gradients in sites of all forest types in China (Table 1). However, the latitudinal patterns observed in these studies are different. Some studies suggest that soil fungi in forest ecosystems follow a similar universal latitudinal trend, as latitudinal changes in temperature are often associated with variations in plant productivity and biodiversity and, thus, may support a higher abundance of soil fungi (2–5). The strongest evidence supporting these studies is that soil fungal richness is negatively correlated across latitudinal gradients globally (6). However, other studies disagree with this view because they found a unimodal trend, with fungal diversity peaking around 40°N in China (7–9) and in the range of 20°N to 50°N in the northern hemisphere (6, 7). The reason for this is that soil fungal diversity is strongly affected by community structure, soil nutrients, and plant-soil interactions, and the combination of these factors results in a unimodal pattern of forest soil fungal latitudinal diversity (7–9). Why are these results different? Here, I attempt to answer this question.

**TABLE 1:** Results of six studies on the variation in fungal biodiversity of forest soils across latitudinal gradients in sites of all forest types across China^*a*^

Reference	No. of sites	Diversity index	Latitudinal pattern	*R* ^2^	*P* value
8	10	Richness	Unimodal	0.764	<0.05
		Shannon index	Unimodal	0.456	<0.05
5	6	Richness	Negative linear	0.648	0.001
3	40	Richness	Negative linear	0.015	<0.001
		Shannon index	Unimodal	0.179	<0.001
		Simpson index	Negative linear	0.143	<0.001
7	28	Richness	Unimodal	0.17	<0.01
4	26	Richness	Negative linear	0.22	<0.001
9	33	Shannon index	Unimodal	0.625	<0.001

a Only significant results are listed.

**FIG 1:**
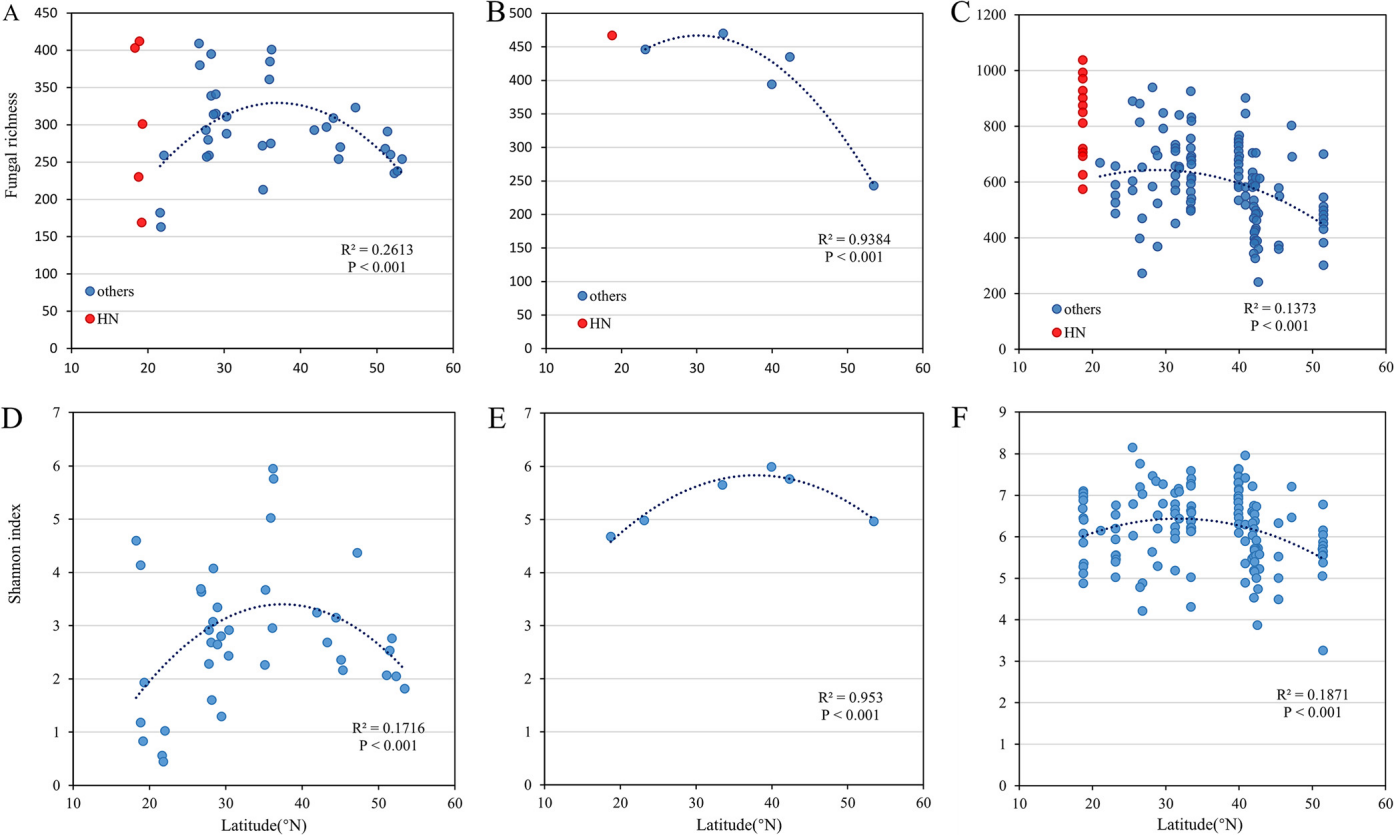
Relationships between latitude and soil fungal richness. Based on data from references 3 (A), 5 (B), and 4 (C). HN refers to samples from Hainan Island. Relationships between latitude and soil fungal Shannon index. Based on data from references 3 (D), 5 (E), and 4 (F).

It is noteworthy that all studies in which negative linear patterns were observed had stations on Hainan Island (Table 1) (3–5, 7–9). If the data collected on Hainan Island were deleted, the trends would change into a unimodal trend owing to the high fungal richness on Hainan Island (Fig. 1A to C). In fact, the environments of continents and islands at the same latitude are quite different, as is the forest soil. Nonmetric multidimensional scaling (NMDS) and principal-coordinate analysis (PCA) showed that the points on Hainan Island were more discrete than those on the other stations (3–5); this showed that the island environment different from the mainland affects the soil fungal community (10, 11). The soil P content of forests on Hainan Island is higher than that of the continental forests at the same latitude, and C, N, P, and their ratios exhibit large spatial variations on the island (12). Nutrient-abundant soil improves microbial nutrition and the competitive advantage of fungi, thus increasing soil fungal richness (13, 14). The diverse habitats with large spatial variations in soil nutrition were also found to be beneficial for soil fungal richness (15). The higher fungal richness on Hainan Island increases the inclination of the trend line and decreases the fungal richness with increasing latitude.

The different categorizations may also result in different results of fungal latitudinal diversity in China. Currently, microbial diversity is mainly calculated using operational taxonomic units (OTUs) based on gene similarity because only a fraction of the global species pool is currently known (16). For this reason, the fungal diversity determined using different criteria shows different latitudinal patterns. In general, ACE and Chao1 richness are frequently used in microbiological studies. However, to study the theory of ecological geography, all six studies have adopted OTU richness or Shannon/Simpson diversity, which are more suitable for large-scale ecological research (3–9). Notably, the studies that show unimodal patterns in the Shannon index also showed unimodal patterns in fungal richness (7–9); however, the studies that showed negative linear patterns in richness showed unimodal patterns in the Shannon index (Fig. 1D to F). In addition, the studies that showed negative linear patterns in richness also showed unimodal patterns in Pielou’s evenness index, which can be used to measure the evenness of fungal communities (see Fig. S1 in the supplemental material). Since Shannon diversity is contributed to by both richness and evenness, OTU richness cannot reveal the role of evenness, which may lead to differences if evenness is ignored (17, 18). Furthermore, the family-OTU correlation of the Shannon index values is better than that of the richness values (Fig. S2). This correlation is similar to the family-species correlation of global terrestrial animal taxa, and it shows that the consistency of the Shannon index in different classification scales is better than the consistency of fungal richness (19). The Shannon index is a function of entropy, which has thermodynamic significance; it can thus better reflect the thermal change caused by latitude (20). Therefore, the fungal latitudinal diversity pattern in China is similar according to the Shannon index but different according to fungal richness.

In conclusion, differences in the results of forest soil fungal latitudinal diversity are caused by the different environments of continents and islands, as well as by the inconsistency between different classification standards. Accordingly, I propose the following three suggestions for future studies of forest soil fungal latitudinal diversity: (i) sites on the mainland and sites on islands should be distinguished in these types of studies, (ii) the Shannon index should be used to represent fungal diversity instead of richness, and (iii) using the diversity of higher taxa (such as family level), instead of OTU level, represents a potential proxy for species-level diversity. Only in these ways can we better describe and understand the forest soil fungal latitudinal diversity in future studies.

### Data availability.

The data have been published in references 3 to 5 and 7 to 9.
