# Supplementary material for: Negative Linear or Unimodal: Why Forest Soil Fungal Latitudinal Diversity Differs across China
Source: Microbiol Spectr. 2023 Feb 22;11(2):e02515-22. doi: 10.1128/spectrum.02515-22 (PMC10100784; doi:10.1128/spectrum.02515-22)
Supplement: Supplemental file 1 — Fig. S1 and S2. Download spectrum.02515-22-s0001.pdf, PDF file, 0.2 MB [file spectrum.02515-22-s0001.pdf]

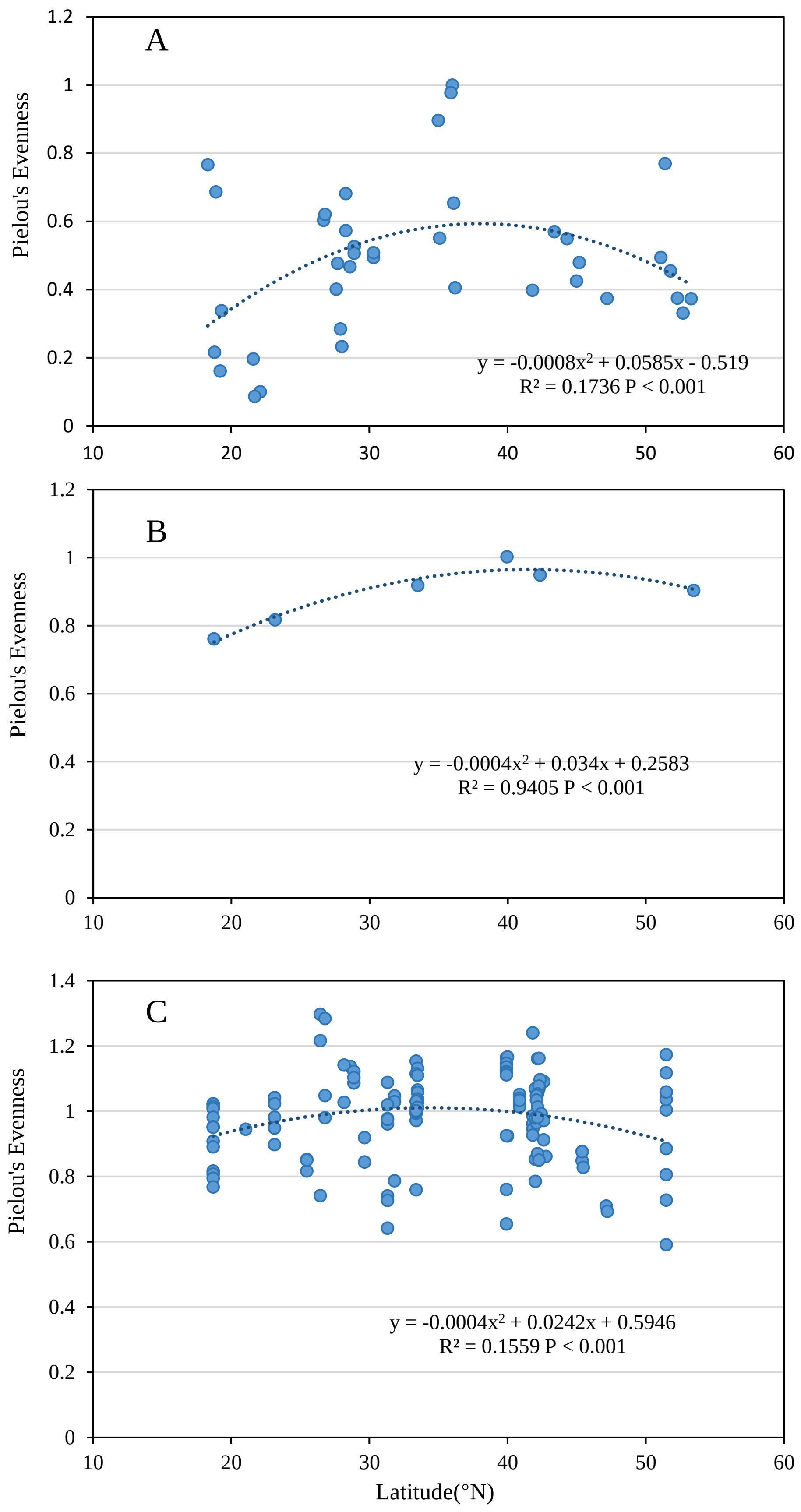

Figure S1. Relationships between latitude and soil Pielou's Evenness, data reference from (A) Hu et al. (2019), (B) Huang et al. (2019), and (C) Liu et al. (2020).

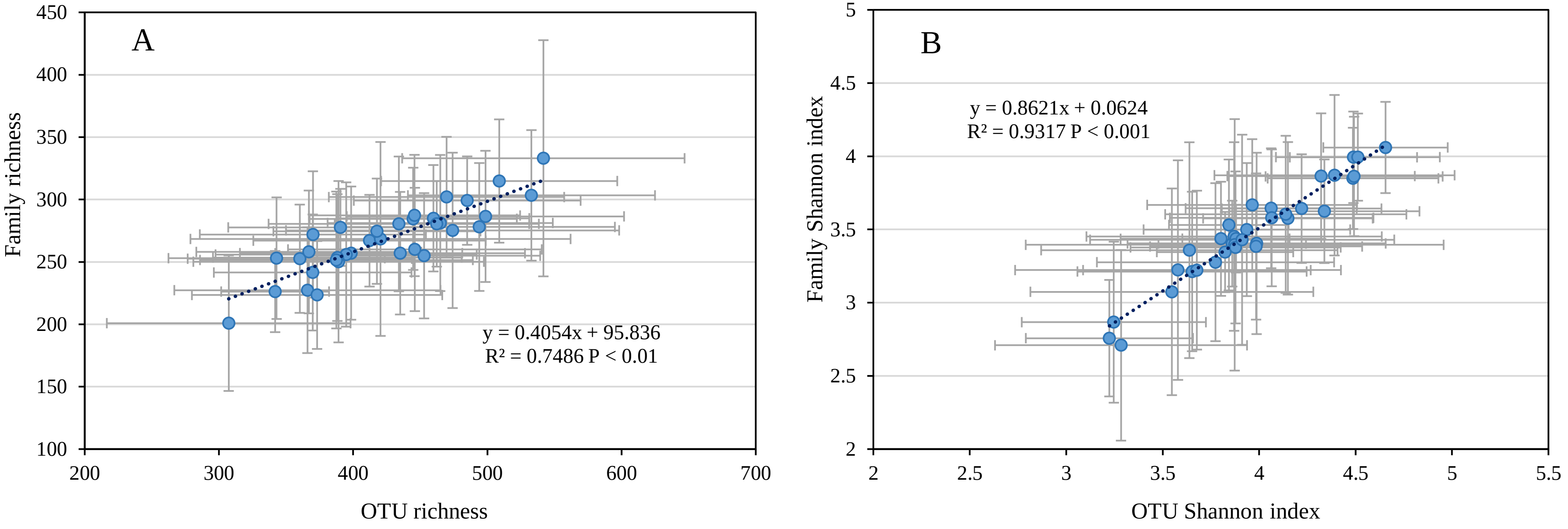

Figure S2. The family–OTU correlation of (A) soil fungal richness, (B) Shannon index. Data from similar sites have been merged.
